# Continuous quality improvement: reducing informed consent form signing errors

**DOI:** 10.1186/s12910-023-00933-w

**Published:** 2023-08-04

**Authors:** Tsui-Wen Hsu, Chi-Hung Huang, Li-Ju Chuang, Hui-Chen Lee, Chih-Shung Wong

**Affiliations:** 1 Institute of Medicine, Superintendent Office and CGHIRB, Cathay General Hospital, Taipei, Taiwan; 2 Department of Cardiology, Cathay General Hospital, Taipei, Taiwan; 3 Cathay General Hospital Nursing Department Supervisor, Cathay General Hospital, Taipei, Taiwan; 4 Department of Anesthesiology, Department of Medical Education and CGHIRB, Cathay General Hospital, Taipei, Taiwan

**Keywords:** Institutional review board (IRB), Informed consent form (ICF), Plan–do–check–act (PDCA), Quality improvement

## Abstract

**Background:**

Adherence to ethical guidelines and regulations and protecting and respecting the dignity and autonomy of participants by obtaining a valid informed consent form (ICF) prior to participation in research are crucial; The subjects did not add signatures next to the corrections made to signatures or dates on the ICF, Multiple signatures in other fields, ICF missing/missing signature, Incorrect ICF version Signed after modification, Correction tape used to correct signature, Impersonated signature, Non-research-member signature, however, ICFs are often not properly completed, which must be addressed. This study analyzed ICF signing errors and implemented measures to reduce or prevent these errors.

**Methods:**

We used the plan–do–check–act (PDCA) cycle to help improve the correctness and validity of ICF signing.

**Results:**

Interim and final reports from January 2016 to February 2020 including 363 ICFs were studied. The total proportion of correct ICF signatures (200, 83.3%) following the PDCA intervention was significantly higher than that before the intervention (P < 0.05). Analysis of the types of signing error demonstrated that signature errors were significantly reduced after the intervention, particularly for subjects did not add signatures next to the corrections made to signatures or dates on the ICF (16, 6.7%) and impersonated signature (0; P < 0.05).

**Conclusions:**

The proportions of other error types—multiple signatures in other fields, missing or unsigned ICF, incorrect signature order, incorrect ICF version, use of correction tape to correct signature, and non-medical profession members signing the ICF—did not differ significantly.

## Background

The main role of an institutional review board (IRB) is to review and oversee clinical research protocols to ensure that all protocols are conducted in accordance with regulations and ethical standards and protect research participants [1, 2]. The missions of an IRB are (1) to educate investigators and research teams on ethical issues, scientific integrity, preventing misconduct, and conflicts of interest and (2) to monitor the implementation of IRB-approved protocols [3, 4]. The main goals of this study was to investigate the effectiveness of utilizing a checklist and face-to-face education intervention to reduce improperly documented consent errors, dynamically revise a standard operating procedure (SOP; Cathay General Hospital Institutional Review Board 2022), and maintain IRB performance regarding oversight of the implementation of all submission protocols [5, 6]. IRBs should review informed consent forms (ICFs) to ensure their validity [7]. Clinical studies must provide an ICF that has been signed and dated by a participant to demonstrate the participant’s willingness to join the study after the investigator has provided a complete and detailed explanation of the study [8]. Obtaining an ICF is an interactive process that requires active engagement with participants to provide comprehensive information to all subjects. The act of signing ICF served as a critical indicator of the participant’s understanding, acceptance and voluntary willingness to participants in the study. Additionally, the absence of a properly signed ICF would raise concerns about potential flaws in the informed consent process, suggesting that participants may not have been provided with all necessary information about the study or might not have enrolled in the study voluntarily [1, 9]. Participants should sign an ICF with sufficient information to make a decision. Investigators must protect and respect the dignity and autonomy of research participants and obtain an ICF before allowing a participant to take part in the study [8, 10].

The essential elements required by three major ethical guidelines and regulations—the Declaration of Helsinki [11, 12], the International Conference on Harmonization (ICH) for Good Clinical Practice (GCP) (ICH GCP 2021), and Taiwan’s Human Subject Research Act (Ministry of Health and Welfare 2011)—are as follows. (1) Prior to an individual’s participation in a trial, an informed consent form should be signed and dated by the participant, their legal representative, or a legally acceptable representative. The investigator or a team member designated by the investigator should obtain ICF signatures from all parties and give the participant or their legally acceptable representative ample time and opportunity to inquire about the details of the trial before signing. (2) All questions about the trial should be answered to the participant or their legal representative. Every ICF should be signed by the participant and the investigator and dated. A copy of the signed ICF should be retained by the investigator, and the original ICF should be kept by the participant. Despite the existence of these regulations, the elements of informed consent are not always followed in letter and spirit. Therefore, principal investigators (PIs) should be educated in how ICFs are correctly signed, and research teams should be properly authorized to execute and explain research content; the participant must clearly and correctly sign the ICF. This is the most important process to ensure the protection of the participant. (3) IRBs should oversee the application of every approved protocol and conduct at least one annual audit.

According to Taiwanese regulations (Ministry of Health and Welfare 2011), the PI is responsible for submitting interim and final reports to the IRB every year to check that the obtainment of valid consent ensures compliance with the participants’ protection and autonomy, that the process is nonthreatening for the participants, and that the relevant provisions for ICF signing are being implemented. The IRB review the ICF signing procedure and identified numerous errors in the signature records in the ICF signing interim and final reports.

From our internal audit in our hospital, we revealed that the research team did not follow regulations and SOPs and obtained invalid ICFs. Therefore, we used a plan–do–check–act (PDCA) intervention to improve quality and reduce errors in ICF signatures and ensure that valid participant consent was obtained. The PDCA cycle is a time-sensitive management model. It was first conceived by Shewhart and later expanded by Dr. Deming in the United States into a quality control cycle that is widely used in management [13, 14].

Quality improvement (QI) is important in IRB operations because it ensures compliance with laws and guidelines and dynamic revision of SOPs to improve IRB oversight of human research activities. QI requires continual efforts by all involved, including PIs, clinical research coordinators, funding institutions, planners, educators, and IRB administrators, to create changes that lead to systematic improvements in performance, professional development, and research outcomes. No studies have yet investigated the reduction of ICF signature errors. This study compared ICF signature errors before and after an educational intervention in our IRB.

## Materials and methods

### Clinical data

This study was divided into two stages. The first stage was before the PDCA intervention, from February 2016 to February 2017; data on 123 ICFs were collected. The data used for analyzing in this study included 33 clinical trials and 90 PI initiated studies in the control group, 23 clinical trials and 218 PI initiated studies in the PDCA intervention group reviewed by our Cathay General Hospital. The second stage was after the PDCA intervention, from February 2018 to February 2020; data on 240 ICFs were collected and defined as the postintervention group.

### Intervention

For the PDCA intervention:

Planning (P). PDCA quality control team was established that included the IRB chair, vice chairpersons, and IRB staff. Improvement of ICF signing was guided by the major ethical guidelines of the ICP GCP (ICH GCP 2021), the Taiwan council Human Subjects Research Act, and IRB SOPs. We developed an ICF signature checklist (Table 1) for PIs and research teams; this checklist emphasized instructions on preventing common errors and correct ICF signing and review of baseline data to identify underlying causes of errors and develop intervention plans.

**Table 1:** Signature of informed consent form checklist

Content	
1. Informed Consent Form (ICF): A written document signed and dated by a participant who voluntarily confirms their willingness to participate in a particular trial after having been informed and having understood all aspects of the trial that are relevant to the participant’s decision to participate.	3*
2. The investigator should obtain the ICF willingly submitted by the participant prior to the beginning of the trial. The investigator or a person designated by the investigator should fully inform the participant of all pertinent aspects of the trial, including the contents of the ICF and documents approved by the Ethics Committee. The investigator or designated person should ensure that the content of the ICF and other trial-related documents is well understood and that the ICF has been signed and dated by the participant.	5 − 1* 5 − 2*
3. All trial staff should be qualified and have the training and work experience necessary to assume their responsibilities in the trial.	14*
4. Prior to the beginning of the trial, the investigator must obtain approval from the Ethics Committee for the ICF and any other written information to be provided to participants. The approval referred to in the preceding paragraph should be made in writing.	16*
5. Any revised ICF and written information shall be approved by the Ethics Committee prior to use; for clinical trials that are approved by a competent authority, the revised documents shall be resubmitted for approval.	17 − 2*
6. Prior to a participant’s participation in the trial, the ICF must be signed and personally dated by the participant, their legal representative, or a legally acceptable representative. Before informed consent may be obtained, the investigator, or a person designated by the investigator, should give the participant or their legally acceptable representative ample time and opportunity to inquire about the details of the trial.	20 − 1* 20 − 2*
7. The sponsor should appoint appropriately qualified individuals for the following tasks: a. to supervise the overall conduct of the trial, b. to handle and verify data, c. to conduct statistical analyses and prepare trial reports, d. to perform operations related to conducting the trial.	54*
8. The investigator should be informed of any CRF (case report form) entry error, omission, or illegibility. The monitor should ensure that appropriate corrections, additions, or deletions are made, dated, explained, and marked with initials by the investigator or by a member of the investigator’s trial staff who is authorized to approve CRF changes for the investigator. This authorization should be documented and filed.	77 − 15*
9. The Ethics Committee should approve studies on the basis of the degree of risk to human participants and continually review ongoing trials at appropriate intervals. These continual reviews should be conducted at least once a year.	87 − 1* 87 − 2*
10. ICF signature order a. Qualified researchers interpreting the participant’s consent form. b. Participant c. Primary investigator/coinvestigator	IRBSOP**

*ICH GCP Article, International Conference on Harmonization of Good Clinical Practice Requirements for ICF (Paraphrased, E6 (R2), 2021)

**Cathay General Hospital Institutional Review Board (IRB) Standard Operating Procedures (SOPs) Version 12 (2022)

Intervention (D). The PDCA quality control staff play a crucial role in training the PIs and research team on the proper procedure for obtaining a signed ICF in accordance with regulatory requirements. Staff emphasize key aspects, including avoiding impersonated signatures impersonated, using the correct version of the ICF, and ensuring that only authorized research members explain the details of the study documented in the ICF. Furthermore, they emphasize the importance of respecting participant’s autonomy and willingness to join the study, In addition to training, the PDCA quality control staff also supervise implementation of the system. First, we implemented a comprehensive approach for ICF signing using a checklist. This involved conducting face-to-face training session with the research team in the IRB office, where step-by-step teaching and feedback was provided. Tests were conducted to evaluate knowledge retention regarding key points. This allowed us to verify the successful implementation of the system and address any questions. To enhance clarity and precision, the ICF signature positions were meticulously divided and prominently marked with eye-catching red, yellow, and blue signs. There visual cues serve as reminders for accurate placement, facilitated differentiation between child and adult ICF, and avoid miss use. Third, we informed the PIs and research teams about the requirement to visit IRB office to obtain a copy of the approved ICF for reference. This visit provided an opportunity to confirm implementation of the relevant processes. In addition, we provided a manual outlining the signing process for the ICF.

Check (C). The PDCA team checked for ICF signing errors, In addition, we regularly re-educated PIs and research teams and released new regulations to solve existing problems and achieve a higher rate of correct ICF signing.

**Fig. 1:**
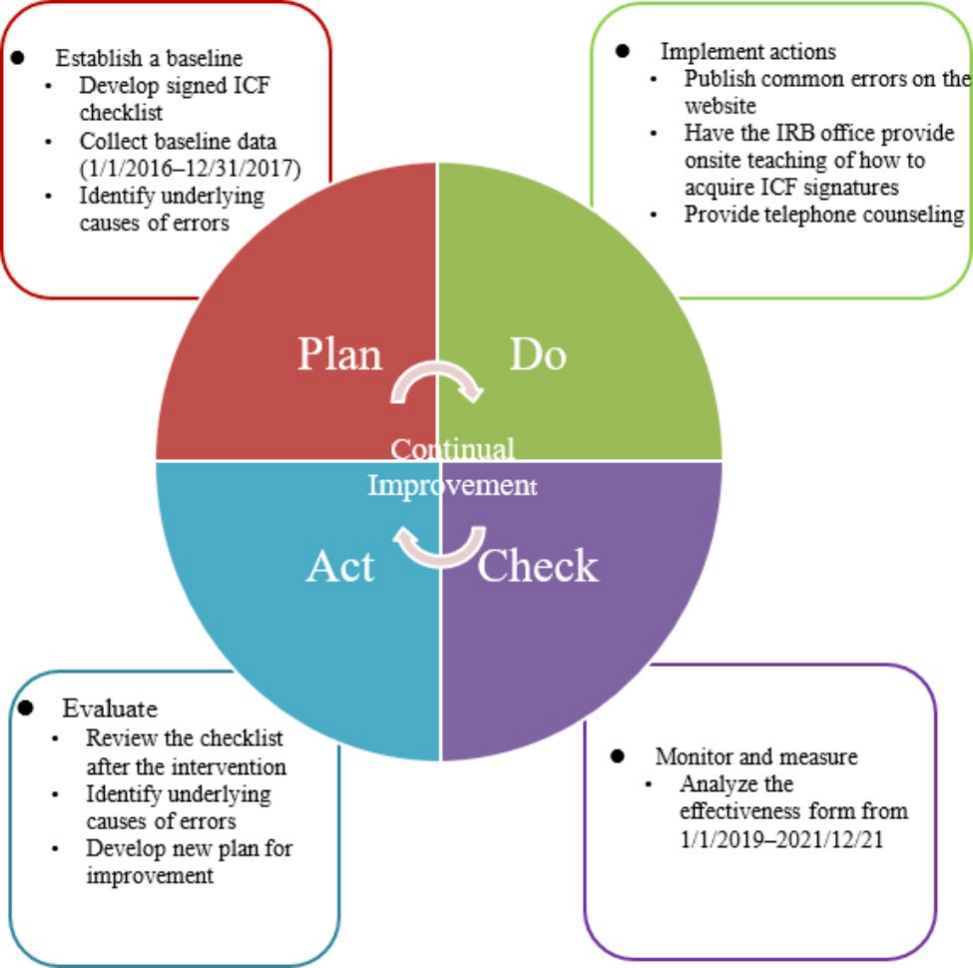
Diagram of PDCA implementation for improving ICF signing quality. Improvement, adjustment and continuous PDCA cycle

Continuously follow up (A). The PDCA team summarized the problems and influencing factors, formulated solutions on the basis of the problems, and incorporated other items to further improve ICF signing before the next PDCA cycle was implemented for continuous and systematic improvement (Fig. 1).

### Statistical methods

Data management and statistical analyses were performed using SPSS version 18 (SPSS Inc., Chicago, IL). Countable data are expressed as number (%). An independent-sample χ^2^ test was employed to make comparisons, with P < 0.05 indicating significance.

### Ethical approval

This QI study was performed with the approval and supervision of the clinical lead of the unit in which the improvement activity was performed. Ethical approval for this study was obtained from the IRB of Taipei Cathay General Hospital (CGH-P111025). This QI study was considered a “minimal risk” study because it only involved the study used existed data collected for other purposes. The data used in this study were collected from our IRB database and data was recorded in deidentified manner.

## Results

From January 2016 to February 2020, the final report and interim report included data on 363 ICFs. Before the intervention, 35 (28.5%) of the ICFs contained errors. our statistical results demonstrated that the rate of correct signing after the PDCA intervention was significantly improved to 83.3% in total 200 ICF forms.

Following the PDCA intervention, the signature error rate reduced by 11. 8%. (Table 2). Signing error type analysis demonstrated significantly fewer signing errors in the post intervention group, in particular, fewer subjects did not add signatures next to the corrections made to the signatures or dates on the ICF (16, 6.7%) and with impersonated signatures (0, 0%; P < 0.05, Table 3). The proportions of other error types—multiple signatures in other fields, missing ICF/ signature, incorrect order of signature, incorrect ICF version, use of correction tape to correct signature, and non-medical profession members signing the ICF—did not differ significantly between the two groups.

**Table 2:** Management scores before and after intervention of ICF writing consultation

Variable	Group		X^2^	P
	Before (N = 123)	After (N = 240)		
			13.194	0.013
Correct signing	88 (71.5%)	200 (83.3%)		

P < 0.05: significant

**Table 3:** ICF error items before and after intervention of ICF writing consultation

Variable	Group		X^2^	P
	Before (N = 123)	After (N = 240)		
Signature error				
The subjects did not add signatures next to the corrections made to signatures or dates on the ICF	21 (17.1%)	16 (6.7%)	9.621	0.003
Multiple signatures in other fields	5 (4.1%)	19 (7.9%)	1.954	0.187
ICF missing/missing signature	6 (4.9%)	10 (4.2%)	0.098	0.790
Incorrect order of signatures	7 (5.7%)	8 (3.3%)	1.141	0.281
Incorrect ICF version	4 (3.3%)	6 (2.5%)	0.172	0.739
Correction tape used to correct signature	1 (0.8%)	3 (1.2%)	0.143	1.000
Impersonated signature	4 (3.3%)	0 (0)	7.892	0.013
Non-research-member signature	2 (1.6%)	1 (0.4%)	1.451	0.266

P < 0.05: significant

Before (Non PDCA intervention from January 2016 to December 2017); After (PDCA intervention from January 2019 to December 2021).

## Discussion

Adhering to correct processes for signing ICFs is an indicator that the rights and interests of participants have been appropriately recognized throughout the research process [15, 16]. This study investigated the quality improvement of ICF signing. Improving the quality of ICF signing is key to improving IRB administration quality and protecting participants’ rights and welfare during a study. PDCA is a continuous QI concept. We analyzed the existing problems with ICF signing, which was critical to improving management. In this study, PDCA was used to improve the ICF signing procedure; this was achieved using a checklist, production of a valid sample ICF by the educational research team, and compliance regulations. Our results demonstrated that the rate of correct and complete ICF signing in the postintervention group was significantly higher than that in the preintervention group; The subjects did not add signatures next to the corrections made to signatures or dates on the ICF was considerably reduced from 21 (17.1%) to 16 (6.7%). This suggests that the PDCA intervention effectively improved quality and reduced ICF signing errors.

ICFs are a crucial ethical and regulatory requirement for clinical research [17, 18]. The quality of informed consent depends on participants’ voluntary affirmation of their understanding, ability, and willingness [19, 20], the process of voluntary decision-making, joint decision-making by the research team, and always acting in the participant’s best interest. Studies have shown that the fulfillment of these conditions guarantees participant willingness [21, 22].

According to the results of a global survey conducted by the Center for Information and Research on Clinical Research Participation in the Asia-Pacific region, 29% of responders said they did not know or were not sure what clinical research was, 69% general public responded that informed consent was difficult to understand, and only 25% were willing to participate in clinical research. A study reported that ICFs are being used as a legal document to protect researchers and sponsors rather than to give participants relevant information for decision-making [21]. Another study demonstrated that distrust was a barrier to study participation for 26% of Indian patients [23, 24]. Insufficient awareness of clinical research information can make participants vulnerable and compromise the quality and effectiveness of the informed consent process [25, 26]. Therefore, it is crucial for research teams to obtain a valid and correctly signed ICF.

ICF information regarding research should be provided by a physician or staff with appropriate scientific training and work experience to fulfill their responsibilities in the trial [7]; in addition, a potential participant’s physical, emotional, and mental capabilities must be considered when obtaining an ICF. An appropriate environment also needs to be provided to ensure the privacy of potential participants. Valid ICFs should be obtained using clear communication between the participant and the research team to gain the participant’s trust through explanation of the research information process. Building awareness of the research team and educating participants about clinical research regulations and ethics are crucial to improving the quality of informed consent obtainment, and strategies are needed to improve the quality of the ICF signing process.

## Conclusions

We adopted PDCA sustainable improvement measures to improve the ICF signing process. We effectively reduced the frequency of ICF signing errors and reduced the rate of ICFs not officially signed after modification. For further improvements to ICF signing, research on how monitoring methods can be improved is necessary.

## Data Availability

The data that support the findings of this study are available from Taipei Cathay General Hospital IRB, but restrictions apply to the availability of these data, which were used under license for the current study and are not publicly available. Data are however available from the authors upon reasonable request and with permission of Cathay General Hospital IRB.
